# Revisiting the white blood cell count: immature granulocytes count as a diagnostic marker to discriminate between SIRS and sepsis - a prospective, observational study

**DOI:** 10.1186/1471-2172-14-8

**Published:** 2013-02-12

**Authors:** Axel Nierhaus, Stefanie Klatte, Jo Linssen, Nina M Eismann, Dominic Wichmann, Jörg Hedke, Stephan A Braune, Stefan Kluge

**Affiliations:** 1 Department of Critical Care, University Medical Center Hamburg-Eppendorf, Martinistr. 52, Hamburg, 20246, Germany; 2 Sysmex Europe GmbH, Bornbarch 1, Norderstedt, 22848, Germany

## Abstract

**Background:**

Sepsis is a serious disease condition and a major cause of intensive care unit (ICU) admission. Its diagnosis in critically ill patients is complicated. To diagnose an infection rapidly, and to accurately differentiate systemic inflammatory response syndrome (SIRS) from sepsis, is challenging yet early diagnosis is vital for early induction of an appropriate therapy. The aim of this study was to evaluate whether the immature granulocyte (IG) count is a useful early diagnostic marker of sepsis compared to other markers. Therefore, a total of 70 consecutive surgical intensive care patients were assessed. IGs were measured from whole blood samples using an automated analyzer. C-reactive protein (CRP), lipopolysaccharide binding protein (LBP) and interleukin-6 (IL-6) concentrations were also determined. The observation period was a maximum of 21 days and ended with the patients’ discharge from ICU or death. Receiver operating characteristic (ROC) analyses were conducted and area under the curve (AUC) was calculated to determine sensitivities and specificities for the parameters.

**Results:**

We found that the IG count significantly discriminates between infected and non-infected patients (*P* < 0.0001) with a sensitivity of 89.2% and a specificity of 76.4%, particularly within the first 48 hours after SIRS onset. Regarding the discriminative power for infection, the IG count was more indicative than other clinical parameters such as CRP, LBP and IL-6, which had a sensitivity of less than 68%. Additionally, the highest diagnostic odds ratio (DOR) with 26.7 was calculated for the IG count within the first 48 hours. During the course of the disease ROC curve analyses showed a superior positive predictive value of the IG count compared to the other measured parameters during the first five days following the fulfillment of SIRS criteria. However, the number of IGs was not correlated with ICU mortality.

**Conclusions:**

The total number of IG in peripheral blood from ICU patients is a good marker to discriminate infected and non-infected patients very early during SIRS. However, the IG count is not suitable as a prognostic marker for mortality. Routine and serial measurement of IGs may provide new possibilities for rapid screening of SIRS patients on ICU with suspected infections.

## Background

In 1992, the American College of Chest Physicians (ACCP) and the Society of Critical Care Medicine (SCCM) established criteria to define and distinguish systemic inflammatory response syndrome (SIRS) and sepsis. According to the ACCP/SCCM consensus conference both SIRS and sepsis are characterized by the occurrence of at least two of the following conditions: (1) body temperature >38°C or <36°C; (2) heart rate >90 beats per minute; (3) respiratory rate >20 breaths per minute or PaCO_2_ <32 mm Hg; (4) white blood cell count >12,000/cu mm, <4000/cu mm, or >10% immature (band) forms. In addition to these criteria, the term sepsis is defined as an inflammatory systemic response arising from an infection [1]. However, this definition of sepsis does not allow accurate staging or prognosis of the host response to infection which is why an expanded list of sepsis markers may reflect the clinical response to infection more appropriately [2].

Despite an increased understanding of the pathophysiology of sepsis and advances in intensive care (ICU) treatment, sepsis still results in considerable morbidity and mortality [3,4]. Early diagnosis in critically ill patients is required to treat inflammation and to avoid the loss of efficacy of targeted anti-infective therapies due to late administration [5]. Good clinical markers should detect infection with high sensitivity and specificity at an early point in time. To date, there is no reliable method of diagnosing sepsis early in the severe syndrome. Microbiological blood cultures are used to identify pathogens but, in addition to their low specificity, they are a poor early marker because of the time needed to obtain results [6,7].

Markers such as interleukin-6 (IL-6), C-reactive protein (CRP) and lipopolysaccharide binding protein (LBP) have been proposed for diagnosing or monitoring sepsis [7,8]. IL-6 is produced by cells of the innate immune system enhancing, as a secreted pro-inflammatory cytokine, early inflammation [9]. Under septic conditions IL-6 is also produced by the endothelium and participates in leukocyte recruitment to organs [10]. Circulating IL-6 levels seem to be correlated with the severity of sepsis [11,12]. An increased IL-6 level also activates the production of acute phase proteins such as CRP and LBP [13,14]. Although CRP has been reported as an indicator of sepsis, several studies have demonstrated increased CRP associated with non-infectious conditions, e.g. major surgery, cardiogenic shock and major trauma [15-17]. LBP is a key participant in the inflammatory response to bacterial infections forming complexes with lipopolysaccharides (LPS) of gram-negative bacteria [18]. Thus, monocytes and macrophages are activated, resulting in the production of pro-inflammatory cytokines and therefore in an enhancement of the clinical presentation of sepsis. However, the correlation between LBP and sepsis remains controversial and findings are contradictory: although elevated LPB concentrations were found to be related to the severity of infection no significant difference could be shown between patients with SIRS and those with sepsis [19,20]. In summary, these biomarkers increase in patients with inflammation due to trauma or surgery, and therefore their diagnostic relevance in critically ill patients with sepsis is far from perfect [21].

The presence of immature granulocytes (IG) in the peripheral blood provides important information indicating enhanced bone marrow activity. Evaluation of the IG count is therefore a promising option in sepsis [22]. In recent studies the appearance of IG in neonatal sepsis, determined with an automated system, was related to mortality [23,24]. In this context, a left shift of granulopoiesis, i.e. an increased number of neutrophil granulocytes or an increased immature/total granulocyte ratio (I/T-ratio), was correlated to sepsis [25].

The present study was conducted to determine the utility of IG# to distinguish between patients with SIRS and those with sepsis.

## Results

### Patients’ characteristics

Seventy consecutive patients fulfilling at least two SIRS criteria [1] were enrolled in the study. Their mean age was 52.4 years (range 19 – 88) and their median ICU length of stay (LOS) was 19.5 days (range 3 – 54). The 28-day, ICU and hospital mortality rates were: 20% (14/70 patients), 27.1% (19/70 patients) and 28.6% (20/70 patients), respectively. Data were analyzed separately for patients without infection (Group 1, n = 19) or with sepsis (Group 2, n = 51). Group 1 consisted of patients where the presence of a primary infectious focus was unlikely (blunt trauma, solid organ malignancy, GI-bleeding), whereas the majority of patients in group 2 had a definitive infectious source (peritonitis, penetrating trauma, ileus [cf. Table 1). Positive blood cultures (group 2) showed a variety of pathogens, including aerobic gram-negative pathogens, aerobic gram-positive pathogens and fungi. There was no difference in ICU mortality between the two groups (26.3% non-infected patients vs. 29.2% infected patients). Also the LOS was not significantly different between survivors and non-survivors in either the non-infected (*P* = 0.052) or infected (*P* = 0.901) groups. To characterize disease severity the SAPS II score was determined on the day of ICU admission. The population had a median SAPS II score of 37 indicating an expected ICU mortality of 19.7% (the observed overall ICU mortality was 27.1%). As expected, the SAPS II score for non-survivors (*n* = 19) was significantly higher than for survivors (*n* = 51) (p = 0.012) with (data not shown). No difference of SAPS II score between survivors and non-survivors was observed in the non-infected or infected patient sub-groups. Patients’ pre-study diagnoses and other characteristics are shown in Table 1.

**Table 1:** Patient characteristics

**Characteristic**	**All**	**No infection**	**Infection**
**Patient numbers**	70	19	51
**Age (yr) (median, range)**	52.9 (19 – 88)	48.0 (21 – 82)	54.4 (19 – 88)
**Male / Female**	51 / 19	17 / 2	34 / 17
**ICU LOS, days**			
**Median (range)**	19.5 (3 – 54)	15.1 (3 – 54)	21.1 (5 – 51)
**95% CI**	17.2 – 21.8	9.2 – 20.9	18.9 – 23.4
**ICU mortality (no.) (%)**	19 (27.1)	5 (26.3)	14 (27.5)
**Pre-study diagnosis**			
**Tumor**	14	5	9
**Trauma**	22	10	12
**Cardiac decompensation**	4	1	3
**Pancreatitis**	5	0	5
**Peritonitis/Mediastinitis**	11	0	11
**Ileus**	5	0	5
**Others**	9	3	6
**Pathogens detected**		*	#
**Gram-positive**		9	19
**Gram-negative**		8	16
**Anaerobic**		0	0
**Viruses**		0	0
**Fungi**		2	8
**Antibiotic treatment**		18	51

Data are given in absolute numbers or median (range) unless specified otherwise (n = 70). Pre study diagnoses with n < 3 are summarized as others. yr = year; ICU = intensive care unit; LOS = length of stay; 95% CI = 95% confidence interval.

*: pathogens in the “no infection” group were isolated at a later stage during the ICU stay and were regarded as colonisation. #: all pathogens in the “infection” group were isolated from blood cultures.

### Superior discriminative power for infection of IG#

**Figure 1:**
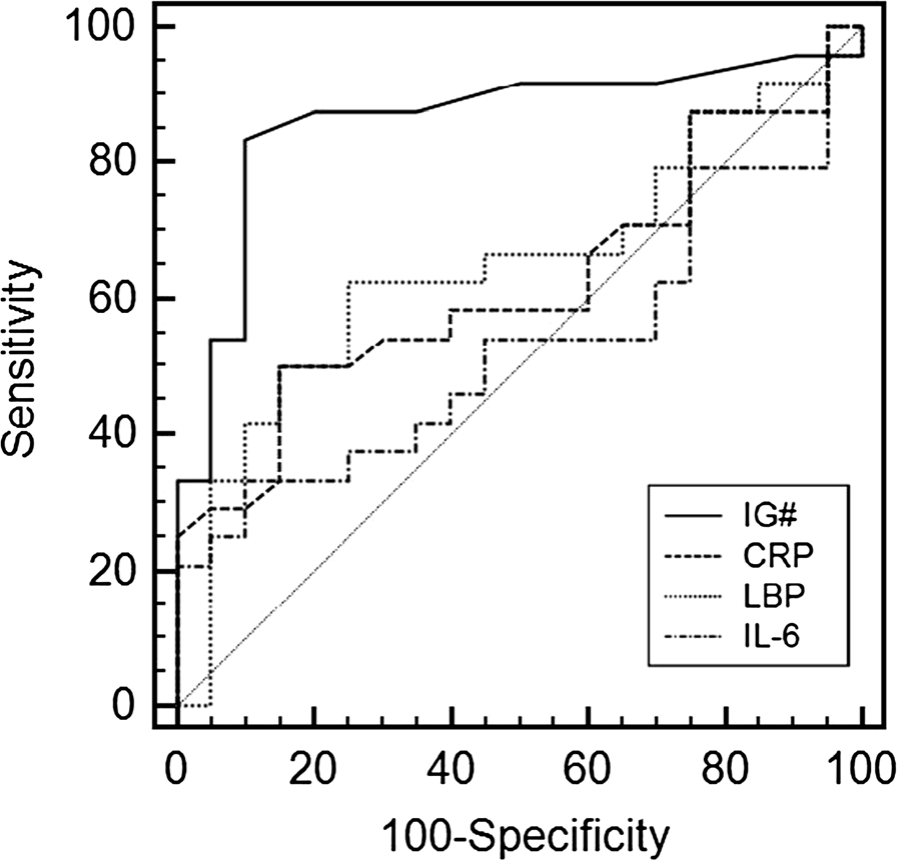
**Diagnostic performance of measured parameters within the first 48 hours.** Receiver operating characteristic (ROC) curves and area under the curves (AUC) were computed to compare the ability of different clinical parameters to predict infection. ROC curves are plotted and corresponding AUC values are given in the table. P < 0.05 was considered a significant difference and is indicated with *. IG#, immature granulocyte count; CRP, C-reactive protein; LBP, lipopolysaccharide binding protein; IL-6, interleukin-6.

ROC analyses were performed to compare the diagnostic performance of four clinical parameters of their predictive potential for sepsis: IG#, CRP, LBP and IL-6. IG# showed a significant distinction between infected and non-infected patients. Thus, a *P* = 0.015 was determined for IG# vs. CRP, *P* = 0.022 for IG# vs. LBP and *P* = 0.003 for IG# vs. IL-6 (data not shown). The superior discriminative power of IG# compared to the other parameters was underlined by its high sensitivity of 89.2% along with a specificity of 76.4% (Figure 1). The highest AUC value of all detected parameters within the first 48 hours after SIRS alert was given by IG# with AUC = 0.861 (*P* < 0.0001). The sensitivities of CRP, LBP and IL-6 did not reach 68% with AUC values below 0.65 (0.529 – 0.648).

Further statistical analysis for independent samples demonstrated the superior discriminative power of IG# over the other parameters (Table 2). Patients with an infection had a significantly higher mean IG# than those without an infection (*P* < 0.0001). Thus, it was possible to distinguish patients with or without infection based on the total number of IGs within the first 48 hours after SIRS criteria had been fulfilled. However, an overlap of IG# was observed between the two groups, with 427.6 +/− 1376.2 cells/μl in patients without an infection and 630.5 +/− 1042.5 cells/μl in patients with an infection. LBP, IL-6 and SOFA were also determined and showed a significant but smaller difference between the two groups. No significant difference for CRP within the first 48 hours was observed between patients with or without infection (Table 2).

**Table 2:** Detection of infection within the first 48 hours

	**Non-infected**	**Infected**	***P*-value**
**IG#** (cells/μl)	427.6 ± 1376.2	630.5 ± 1042.5	<0.0001***
**CRP** (mg/l)	132.1 ± 73.6	170.5 ± 104.3	0.077
**LBP** (μg/ml)	61.9 ± 44.3	81.8 ± 46.9	0.048*
**IL-6** (ng/l)	457.2 ± 691.7	1789.9 ± 9514.0	0.011*
**SOFA** (95% CI)	9.1 (8.2 – 9.9)	7.9 (7.3 – 8.5)	0.024*

Clinical parameters of patients with or without infection were determined and the P-value between these two groups was calculated using Student’s *t* test (CRP, LBP and SOFA) or Mann–Whitney *U*-test (IG# and IL 6); P < 0.05*; P < 0.005**; P < 0.0005*** (n = 70). Data are given as mean ± SD or mean score value (SOFA). IG#, immature granulocyte count; CRP, C-reactive protein; LBP, lipopolysaccharide binding protein; IL 6, interleukin-6; SOFA, sequential organ failure assessment score.

In order to determine the dynamic response to sepsis, the parameters were further analyzed over the course of 21 days after the initial SIRS alert. Five periods were considered; period 1 (days 1–2), period 5 (days 3–5), period 10 (days 6–9), period 15 (days 10–14), and period 20 (days 15–21). The mean value for each period was calculated for each parameter. Since data did not follow a normal distribution, the Mann Whitney *U*-test was used for further analysis.

**Figure 2:**
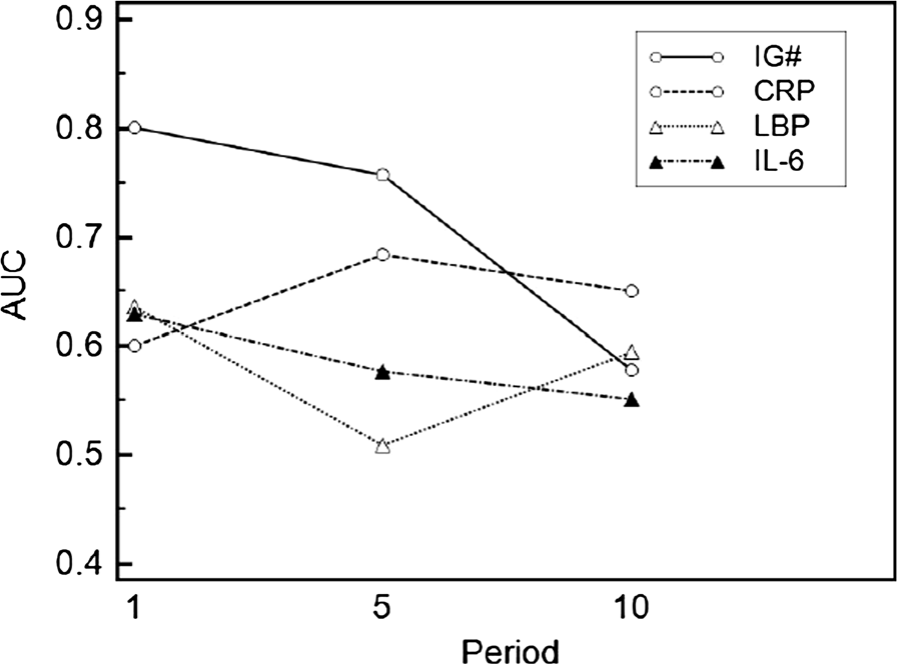
**Prediction of infection.** Predictive value of clinical parameters for infection was computed using receiver operating characteristic (ROC) curves and area under the curve (AUC). Parameter differences between non-infected vs. infected patients were assessed using Mann–Whitney *U*-test; significance is quoted as P < 0.05*; P < 0.005**; P < 0.0005*** (n = 70). Positive predictive value [PPV] and negative predictive value [NPV] were calculated for each period; infection prevalence of 55.71% in period 1, 67.14% in period 5 and 71.43% in period 10. AUC values are plotted against study period and numerical data of AUC [PPV / NPV] are given in the table. IG#, immature granulocyte count; CRP, C-reactive protein; LBP, lipopolysaccharide binding protein; IL-6, interleukin-6; SOFA, sequential organ failure assessment score.

The five clinical parameters, IG#, CRP, LBP, IL-6 and SOFA score were analyzed for their discriminative power to diagnose infection (Figure 2). IG# showed a highly significant difference between non-infected and infected patients early in period 1 and period 5, (*P* < 0.0001). The same was true for LBP, IL-6 and SOFA score in period 1, but not for CRP. The superior predictive value of IG# towards the other measured parameters was underlined by its high positive and negative predictive values, particularly in periods 1 and 5.

Additionally, the highest diagnostic odds ratio (DOR) was calculated for IG# in period 1 with 26.7 (Table 3). For all other clinical parameters a DOR of 3.8 was not exceeded in period 1. By period 5 and 10 the DOR for IG# had decreased to 4.8.

**Table 3:** Diagnostic odds ratio for infection

	**Period 1 (d 1–2)**	**Period 5 (d 3–5)**	**Period 10 (d 6–9)**
**IG#**	26.7 [89.2 / 76.4]	7.9 [81.6 / 64.1]	4.8 [27.9 / 92.6]
**CRP**	3.8 [42.9 /83.3]	8.3 [89.2 / 50]	10.0 [66.7 / 83.3]
**LBP**	3.6 [67.3 / 63.9]	2.5 [17.6 /92]	6.9 [27.7 / 94.7]
**IL-6**	3.0 [54.5 / 71.4]	2.0 [48.4 / 68.5]	2.6 [32.4 / 84.6]
**SOFA**	3.7 [71.8 /59.0]	1.8 [65.4 / 48.3]	2.7 [81.8 / 37.2]

Diagnostic odds ratio (DOR) for infection was calculated from the sensitivities and specificities for clinical parameters. DOR [sensitivity% / specificity%] are given in the table for each period. IG#, immature granulocyte count; CRP, C-reactive protein; LBP, lipopolysaccharide binding protein; IL-6, interleukin-6; SOFA, sequential organ failure assessment.

### Prognostic power for mortality of IG# in late stages

**Figure 3:**
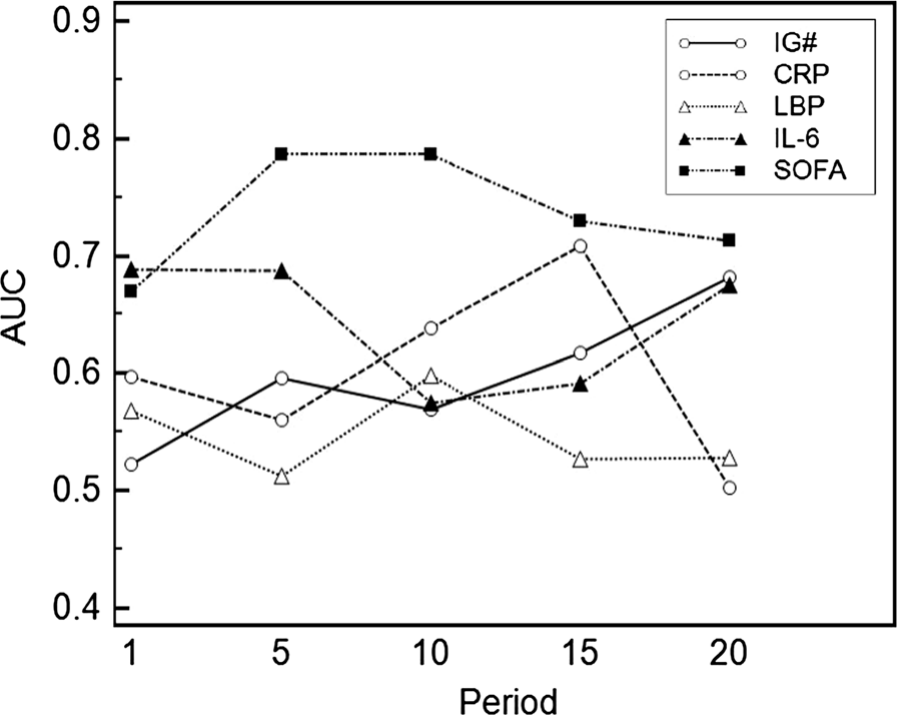
**Prediction of ICU mortality.** The predictive value of clinical parameters for ICU mortality was computed using receiver operating characteristic (ROC) curves and area under the curve (AUC). Parameter differences between survivors and non survivors were assessed using Mann–Whitney *U*-test; significance is quoted as P < 0.05*; P < 0.005**; P < 0.0005*** (n = 70). AUC values are plotted against study period and numerical values are given in the table. IG#, immature granulocyte count; CRP, C-reactive protein; LBP, lipopolysaccharide binding protein; IL 6, interleukin-6; SOFA, sequential organ failure assessment score.

We also assessed the prognostic sensitivity and specificity of each characteristic with respect to ICU mortality. AUC values of each individual parameter are presented in (Figure 3). We found that the SOFA-score significantly differentiated between survivors and non-survivors during the whole ICU stay with AUC values of 0.670 up to 0.786 (*P* = 0.0004 to <0.0001). IL-6 also provided significant discrimination on almost all days with AUC values of 0.592 to 0.688 (*P* < 0.05 to <0.0001), except in period 10. Except for a significant AUC value of 0.708 (*P* = 0.037) for CRP in period 15, CRP did not predict ICU mortality. A significant difference between survivors and non-survivors was detected for IG# in period 15 (AUC = 0.617, *P* = 0.042) and period 20 (AUC = 0.682, *P* = 0.0001). In contrast, LBP was not correlated with ICU mortality.

## Discussion

We have demonstrated that the IG count discriminates between SIRS patients with and without infection. We observed a significant difference within the first five days after the initial SIRS alert, and particularly within the first 48 hours (*P* < 0.0001). Polymorphonuclear neutrophil granulocytes (PMN) are the first line effectors of host defense against bacteria and other pathogens representing about 50% to 60% of all circulating white blood cells (WBC) [26]. Induced by granulocyte-colony stimulating factor (G-CSF), PMN develop from progenitor cells and maturate in the bone marrow over several stages into mature segmented neutrophils [27]. After a maturation period of seven to ten days they migrate into the peripheral blood. Healthy individuals do not have immature granulocytes present in their peripheral blood and consequently the incidence of IG in the periphery indicates substantially increased bone marrow activation as seen in sepsis. Besides the microbiological evidence for infection there are no other generally accepted accurate clinical parameters defined by the ACCP/SCCM Consensus Conference Committee to distinguish sepsis from SIRS so far. Therefore we investigated changes in immature granulocyte numbers in patients with infections. In a previous study, Selig *et al.* found significantly elevated numbers of myeloid progenitor cells in patients with bacterial infections compared with healthy controls [28]. In another study, Ansari-Lari *et al.* found a significantly higher percentage of immature granulocytes in infected than in non-infected patients and designated a percentage of IG of more than three (IG% > 3) as a predictor of sepsis, with a specificity of more than 90% [23]. In that study patients were enrolled who were admitted to the Emergency Department or already were hospitalized and IG% was measured over five consecutive days. In the present study the focus was on patients who were admitted to the ICU and IGs were measured over twenty-one consecutive days. In line with these previous findings, our study confirms that the absolute number of immature granulocytes is significantly higher in infected patients than in non-infected controls. We also demonstrated that the IG count had greater sensitivity and specificity (89.2% and 76.4% respectively) than other parameters (CRP, LBP and IL-6) which all had sensitivities of less than 68%.

We also compared the performance of the IG count with CRP, LBP and IL-6, which are parameters commonly evaluated in patients with suspected infection or sepsis. Although all parameters except CRP were significantly increased in infected patients, the highest discriminative power for infection in the first 48 hours was shown by the total number of immature granulocytes (*P* < 0.0001). ROC analyses showed the highest area under the curve (AUC) for the IG count together with the highest sensitivity (89.2%). Only the AUC for IGs showed significance compared to all other clinical parameters in the first 48 hours.

In later periods the absolute numbers of IGs showed the highest significance for positive prediction of infection during period 5 (days 3–5). While LBP and IL-6 levels were not meaningful, the CRP level was significantly elevated. Synthesis of the acute phase protein CRP is activated by the pro-inflammatory cytokine IL-6 [29], which is in accordance with our findings since we observed a significant discriminatory power of CRP only during period 5 whereas elevated IL-6 levels occurred within the first 48 hours. By period 10 (days 6–9) none of the evaluated parameters were meaningful and no significant difference in the AUC of different parameters could be observed. This may have been due to beneficial effects of early antibiotic treatment given to almost every patient suspected of having an infection. We conclude that the IG count displays superior discriminative power for infection over CRP, LBP and IL-6, particularly within the decisive first 48 hours after the onset of SIRS.

In addition, for the first 48 hours a high diagnostic odds ratio (DOR) for infection of 26.7 could be calculated for the IG count [30]. All other parameters such as CRP, LBP and IL-6, had a DOR lower than 10 at any point in time.

Secondly, we examined the ability of IG count, CRP, LBP, IL-6 and the SOFA score to predict ICU mortality. As expected, the SOFA score, which is well-known and widely used as a predictor of mortality in patients with severe sepsis [31,32], showed the greatest significant correlation to ICU mortality during the entire observation period. A significant differentiation between survivors and non-survivors was obtained from IL-6 levels in periods 1 and 5 but surprisingly also at later stages during periods 15 (days 10–14) and 20 (days 15–21), as well. In early stages a correlation of high IL-6 levels with mortality in critically ill patients is well-known [33,34]. However, the correlation is not usually sustained and IL-6 is considered to have a short half-life. The significant values observed even during periods 15 and 20 in the present study may be explained by the increasing relative number of infected patients during these periods because of the earlier discharge of non-infected patients from the ICU. Thus, the balance of the two examined groups is skewed towards the infected population. The significant values of the IG count for periods 15 and 20 may be interpreted in the same manner. Therefore, the IG count may not have prognostic power for mortality as is the case for CRP and LBP. For CRP these findings are consistent with the literature where no correlation was observed between CRP concentrations and the severity and mortality of sepsis [35]. The prognostic power of LBP for the occurrence of sepsis is controversial. In one study the LBP level was significantly elevated in non-survivors vs. survivors on days 2 and 7 [36]. In contrast, although a higher LBP concentration was found in infected than in non-infected patients, a correlation between LBP and sepsis mortality could not be detected in another study [19].

Recently, many potential biomarkers to identify sepsis and predict disease outcome have been examined. In particular, acute phase proteins such as procalcitonin (PCT) have been investigated. PCT is now used as a supportive clinical parameter to clarify diagnosis, but due to its high negative predictive value (99%) it is more frequently used to rule out sepsis [37]. A comparison of the IG count with PCT in terms of positive predictive values for sepsis should be considered for future studies.

While our findings suggest that the IG count is not suitable as a prognostic marker for mortality it is a good and useful early marker for distinguishing infected and non-infected patients. Our study population included a wide range of patients with diverse causes for ICU admission and various disease stages. The distinct correlation of the IG count with the presence of infection shows that the former parameter is representative in general and not only valid for a special patient subgroup. The IG parameter on the XE-2100 has been previously validated [22]. In this study, the automated IG count was compared to routine manual microscopy using the National Committee for Clinical Laboratory Standards (NCCLS) document H20-A [38], resulting in very good agreement between the two methods with a correlation coefficient of *r* = 0.9 [22]. In addition, reference values for IGs measured in the “differential channel” were generated and a 5^th^ percentile of zero and a 95^th^ percentile of 0.03x10^9^/L with neither gender nor age dependency was detectable [39]. Taken together, the automated IG count was found to be superior to manual microscopy whilst providing fast, inexpensive, accurate and reliable quantification, particularly for very small proportions with counts less than 5% of total WBC [40]. Our findings are in line with a recent work by Mardi *et al.*[41] who also demonstrated the ability of white cell morphology to predict sepsis.

In summary, we have shown that, within the first 48 hours after the first SIRS alert, an elevated number of immature granulocytes indicates infection. Measuring the IG count with a fully automated hematology analyzer provides new possibilities for rapid and early screening of SIRS patients on the ICU with suspected infections.

## Conclusions

Our findings demonstrate that sepsis is associated with an increased immature granulocyte count. The IG count can differentiate between patients with an infection and those who are not infected, particularly within the first critical hours after an initial SIRS alert. Using ROC analysis we found the IG count a superior biomarker for sepsis compared to C-reactive protein, lipopolysaccharide binding protein and interleukin-6.

## Methods

### Study design

Following approval by the local Ethical Committee who had waived the need for informed consent a total of 70 consecutive patients were included. The study population consisted of patients aged between 18 and 90 years, who were admitted to the surgical ICUs of the University Medical Center Hamburg-Eppendorf and who developed signs of SIRS within 48 hours of admission. Exclusion criteria were: malignancy, immunosuppressive or immunostimulatory therapy, and recent organ transplantation. The observation period was a maximum of 21 days and ended with the patients’ discharge from ICU or death.

For statistical analysis the patients were classified as those with or without infection according to the ACCP/SCCM Consensus Conference criteria. Group 1 included patients without an infection; Group 2 included those with an infection. Infection was diagnosed either by blood culture or microbiological tests from catheters, sputum specimens, intraoperative smears, etc.

Four parameters, IG#, CRP, LBP and IL-6, were analyzed especially within the first 48 hours because of their predictive potential for sepsis.

### Determination of immature granulocyte counts

White blood cell count, percentage of immature granulocytes (IG%), and absolute immature granulocyte count (IG#) were determined in blood samples using an automated hematology analyzer (XE 2100, Sysmex, Kobe, Japan). The method is described in detail elsewhere [23]. In short, the IG count includes promyelocytes, myelocytes and metamyelocytes and is performed in differential channels of the analyzer. A specific surfactant induces hemolysis of erythrocytes and the formation of ultramicroscopic pores in the leukocyte cell membrane. The increased permeability of leukocytes allows a polymethine dye to enter the cells with high affinity for nucleic acid. Subsequently, the cells are analyzed by nucleic acid fluorescence and side scatter.

### Determination of other parameters

CRP was measured with a routine turbidimetric assay (Roche Diagnostics on Hitachi Modular). Determination of LBP was performed by immunoassay (DPC Biermann GmbH, Bad Nauheim, Germany) and IL-6 concentrations were measured in serum using an immuno-luminometric assay (CLIA) (DPC Biermann GmbH, Bad Nauheim, Germany).

Simplified Acute Physiology Score II (SAPS II) [42] was obtained within the first 24 hours of ICU admission and Sequential Organ Failure Assessment (SOFA) score [43] was calculated daily.

### Statistical methods

Data were analyzed using MedCalc for Windows, version 11.3.3.0 (MedCalc Software, Mariakerke, Belgium). The Kolmogorov-Smirnov test was used to verify the normality of distribution of continuous variables, with *P* > 0.05 considered as non-significant and thereby normally distributed. Differences between groups were assessed using Student’s *t*-test for normally and Mann–Whitney *U*-test for non-normally distributed variables. Computation of dichotomous target values was performed by *χ*^2^ test. Receiver operating characteristic (ROC) analyses were conducted and the area under the curve (AUC) was calculated from the sensitivities and specificities for clinical parameters. In addition, diagnostic odds ratios (DOR) were computed; defined as [sensitivity/(1-sensitivity)] / [(1-specificity)/specificity]. The DOR can be read as the ratio of the odds of disease with a positive test relative to the odds of disease with a negative test. A test is characterized as useless with a DOR of 1, whereas a DOR higher than 25 represents a useful test and a DOR higher than 100 characterizes a good test [30].

## Competing interest

AN and SK have received an unrestricted research grant by Sysmex Europe. SK and JL are scientific employees of Sysmex Europe, Norderstedt, Germany. All other authors declare that they have no competing interests.

## Authors’ contributions

AN designed and coordinated the study and co-drafted the manuscript. SK participated in the performance of statistical analyses and in preparing the manuscript. NME and JH contributed to data acquisition and carried out the blood measurements and immunoassays. JL participated in the design of the study and coordination and performed the statistical analyses. DW, SAB and SK participated in study design, drafting of the manuscript and coordination of the study. All authors read and approved the final manuscript.
